# Characterization of the Phytochemical Constituents of Taif Rose and Its Antioxidant and Anticancer Activities

**DOI:** 10.1155/2013/345465

**Published:** 2013-10-27

**Authors:** El-Sayed S. Abdel-Hameed, Salih A. Bazaid, Mahmood S. Salman

**Affiliations:** ^1^Natural Products Analysis Laboratory, Faculty of Science, Taif University, P.O. Box 888, Taif-Alhaweih 21974, Saudi Arabia; ^2^Laboratory of Medicinal Chemistry, Theodor Bilharz Research Institute, P.O. Box 30, Imbaba, Giza, Egypt

## Abstract

Ward Taifi (Taif rose) is considered one of the most important economic products of Taif, Saudi Arabia. In this study both fresh and dry Taif rose were biologically and phytochemically investigated. The 80% methanol extracts and *n*-butanol fractions of dry and fresh Taif rose had high radical scavenging activity toward artificial 1,1-diphenyl picrylhydrazyl (DPPH)^•^ radical with SC_50 _values range 5.86−12.24 *µ*g/ml whereas the aqueous fractions showed weak activity. All samples had *in vitro* anticancer activity toward HepG2 with IC_50_ < 20 *µ*g/ml which fall within the criteria of the American Cancer Institute. High positive correlation appeared between the antioxidant activity and total phenolics whereas there is no correlation between total phenolics and anticancer activity. The LC-ESI(− ve)-MS analysis of all extracts indicate the presence of phenolic compounds belonging to hydrolysable tannins and flavonol glycosides. In conclusion, the presence of this is considered to be the first phytochemical report that identifies the major compounds in dry and fresh roses using HPLC-ESI-MS. The methanol extracts and its *n*-butanol and aqueous fractions for both fresh and dry Taif rose could be used as preventive and therapeutic effective natural agents for diseases in which free radicals involved after more *in vitro* and *in vivo* studies.

## 1. Introduction 

The use of natural resources especially plants increases daily for the discovery of new therapeutic agents. Medicinal plants are the richest bioresource of drugs in traditional medicines, modern medicines, nutraceuticals, food supplements, folk medicines, pharmaceutical intermediates, and chemical entities for synthetic drugs. Natural products from plants continue to be used in pharmaceutical preparations as crude extracts, fractions, or pure compounds. Several active compounds have been discovered in plants and used directly as patented drugs like Taxol, Artemisinin, and Maprouneacin [1, 2]. Many studies have proven to be that reactive oxygen species (ROS), and free radicals play a vital role in maintaining human health. When the balance between the generating and scavenging of ROS and free radicals *in vivo* is destroyed, an oxidative stress would happen, which might lead to extensive oxidative damage to cellular biomolecules, such as DNA, proteins, and lipids. Many chronicdiseases such as hyperlipidemia, hyperpiesia, and cancer have proved to be associated with the existence of oxidative stress [3, 4]. In recent years, several dietary and herbal formulations that have free radical scavenging potential have gained attention in treating such chronic diseases. In spite of the strong radical scavenging activity of synthetic antioxidants, they usually have side effects. Thus the interest in finding natural antioxidants, without undesirable side effects, has increased greatly. The antioxidative phytochemicals especially phenolic compounds found in vegetables, fruits, and medicinal plants have received increasing attention for their potential role in prevention of human diseases [1].

*Rosa* genus (family Rosaceae) is an important ornamental plant and has been referred to as the queen of flowers. *Rosa* genus contains over 150 species that are widely distributed in Europe, Asia, Middle East, and North America. Rose is one of the most important crops in the floriculture industry and is used as cut flowers, potted plant, and garden plants. Rose products have also been used in the food, perfumery, and cosmetics industries for many years [4–6]. *Rosa damascena* Mill is one of the most important *Rosa* species. This plant is called Damask rose because it was originally brought to Europe from Damascus [7]. The main products of Damask rose are rose oil, rose water, rose concrete, rose absolute, and dried petals, and these products are used in perfume, cosmetic, pharmaceutical, and food industries [5, 8, 9]. Flowers of Damask rose were reported to have astringent, analgesic, anti-inflammatory, antidepressant, antibacterial, diuretic, and anti-HIV activity, and they are used in folk medicine as a mild laxative [10–12].

Taif rose, Ward Taifi (*Rosa damascena trigintipetala *Dieck), is a type of Damask rose which is considered one of the most important economic products of Taif governorate, Saudi Arabia. In this study, the fresh and dry roses were extracted with 80% methanol followed by partitioning the aqueous solution of extracts with *n*-butanol. The crude 80% methanol extracts, *n*-butanol, and aqueous fractions were phytochemically and biologically investigated. The biological investigations included *in vitro* antioxidant and anticancer activity. The total phenolics, flavonoids, and flavonols were estimated, in addition to analysis by hyphenated techniques including high performance liquid chromatography coupled by electrospray ionization mass spectrometry (HPLC-ESI-MS).

## 2. Materials and Methods

### 2.1. Chemicals

All solvents, standards, and reagents are analytical and HPLC grade. 1,1-diphenyl picrylhydrazyl (DPPH)^•^ free radical and Folin-Ciocalteu's reagent (FCR) are from Fluka Chemicals. Aluminum chloride, sodium carbonate, sodium phosphate, ammonium molybdate, ascorbic acid, petroleum ether, ethyl acetate, methanol, ethanol, acetic acid, trichloroacetic acid, formic acid, sulphuric acid, sulphorhodamine-B (SRB), catechin, taxifolin, rutin, quercetin 3-O-*β*-D-glucoside, kaempferol 3-O-*β*-D-glucoside, kaempferol 3-O-*α*-rhamnoside, quercetin 3-O-*α*-rhamnoside, quercetin, and apigenin are from Sigma-Aldrich Chemicals. Solvents for LC-MS (methanol and acetonitrile) are HPLC grade from Sigma-Aldrich Chemicals.

### 2.2. Collection of Roses and Preparation of Different Extracts and Fractions

During harvest season (March–April 2012), Taif roses were collected from rose farms in the El-Hadda region, Taif governorate, Saudi Arabia. After removing the green parts of roses, part of them was used fresh, and another part was dried on shade air-dried place and powdered using an electric mill. Known weights of cut fresh, and dry powdered rose (900 and 300 g, resp.) were extracted three times by 80% methanol: 4 L for fresh rose and 2 L for dry rose. The solvent was removed under vacuum using rotary evaporator affording known weight of each 80% methanol extract (112 and 97 g). Thirty grams of each 80% methanol extract were dissolved in 150 mL distilled water and partitioned with *n*-butanol (3 × 150 mL solvent). The organic and aqueous layers were separated, collected, and evaporated under vacuum using rotary evaporator affording known weight of each respective fraction (9.35 and 10.54 g for *n*-butanol fraction; 18.65 and 17.27 g for aqueous fractions). The 80% methanol extracts, *n*-butanol, and aqueous fractions were stored in brown glass bottles and become ready for investigation.

### 2.3. Antioxidant Activity

Three different chemical methods were used for evaluating the antioxidant activity of crude methanol extracts, *n*-butanol, and aqueous fractions, 1,1-diphenyl picrylhydrazyl scavenging activity, phosphomolybdenum method, and reducing power assay. These assays were performed as described by Abdel-Hameed et al., 2012 [1].

#### 2.3.1. Scavenging Ability towards 1,1-Diphenyl Picrylhydrazyl (DPPH)^•^ Radical

This method depends on the reduction of purple DPPH radicals to a yellow coloured diphenyl-picrylhydrazine, and the remaining DPPH radicals which show maximum absorption at 517 nm were measured using UV-Vis spectrophotometer (Jenway 6405). Two mL of different concentrations of each sample were added to 2 mL solution of 0.1 mM DPPH. An equal amount of methanol and DPPH served as control. After 20 min of incubation at 37°C in the dark, the absorbance was recorded at 517 nm. The experiment was performed in triplicates. The DPPH radical scavenging activity was calculated according to the following equation:

(1) %  DPPH  radical  scavenging  activity  =[1−(AsampleAcontrol)]  ×100,

where *A*_sample_ and *A*_control_ are absorbance of the sample and control. The SC_50_ (concentration of sample required to scavenge 50% of DPPH radicals) values were also determined. 

#### 2.3.2. Determination of the Total Antioxidant Capacity by Phosphomolybdenum Method

The assay is based on the reduction of Mo (VI) to Mo (V) by the antioxidants and subsequent formation of a green phosphate/Mo (V) complex at acid pH. 300 *μ*L of each sample solution and ascorbic acid (100 *μ*g/mL) were combined with 3 mL of reagent (0.6 M sulfuric acid, 28 mM sodium phosphate, and 4 mM ammonium molybdate). A typical blank solution containing 3 mL of reagent solution and an appropriate volume of the same solvent was used for the sample. All tubes were capped and incubated in a boiling-water bath at 95°C for 90 min. After the samples were cooled to room temperature, the absorbance of each sample was measured at 695 nm against the blank using a UV/Vis spectrophotometer. The experiment was performed in triplicates. The antioxidant activity was expressed as the number of equivalents of ascorbic acid.

#### 2.3.3. Reducing Power Assay

Two mL of each sample and ascorbic acid in methanol (200 *μ*g/mL) were mixed with 2 mL of sodium phosphate buffer (0.2 M, pH 6.6), and 2 mL of 1% K_3_Fe(CN)_6_ were incubated at 50°C for 20 min. After adding 2 mL of trichloroacetic acid, the mixture was centrifuged at 3000 rpm for 10 min. The supernatant solution (2 mL) was taken out and immediately mixed with 2 mL of methanol and 0.5 mL of 0.1% ferric chloride. After incubation for 10 min, the absorbance against the blank was determined at 700 nm UV/Vis spectrophotometer. Triplicates were made for each tested sample and ascorbic acid. The increase in absorbance of the reaction mixture indicates an increased reduction power. The reducing power activity was expressed as the number of equivalents of ascorbic acid.

### 2.4. Anticancer Activity

The crude methanol extracts, *n*-butanol, and aqueous fractions were investigated *in vitro* toward human liver carcinoma cell line (HepG2) (obtained frozen in liquid nitrogen (−180°C) from the American Type Culture Collection and were maintained in the National Cancer Institute, Cairo, Egypt, by serial subculturing), using the method of Skehan et al. (1990) [13] at the National Cancer Institute in Egypt. This is a colorimetric assay that estimates cell number indirectly by staining total cellular protein with the dye sulphorhodamine-B (SRB). This dye is a bright pink aminoxanthrene dye with two sulphonic groups. It is a protein stain that binds to the amino groups of intracellular proteins under mildly acidic conditions to provide a sensitive index of cellular protein content. Cells were seeded in 96-well microtiter plates at a concentration of 5 × 10^4^-10^5^ cell/well in a fresh medium and left to attach to the plates for 24 h. For each sample, different concentrations (0, 5, 12.5, 25, and 50 *μ*g/mL) were added to wells. Wells were completed to total of 200 *μ*L volume/well using fresh medium and incubated for 48 h at 37°C in 5% CO_2_. Following 48 h treatment, the cells were fixed with 50 *μ*L cold 50% trichloroacetic acid for 1 h at 4°C. Wells were washed 5 times with distilled water and stained for 30 min at room temperature with 50 *μ*L 0.4% SRB dissolved in 1% acetic acid. The wells were then washed 4 times with 1% acetic acid. The plates were air-dried, and the dye was solubilized with 100 *μ*L/well of 10 mM tris base (pH 10.5) for 5 min on a shaker (Orbital Shaker OS 20, Boeco, Germany) at 1600 rpm. The optical density (O.D.) of each well was measured spectrophotometrically at 564 nm with an ELIZA microplate reader (Meter tech. Σ 960, USA). The mean background absorbances were automatically subtracted, and mean values of each drug concentration were calculated.

The experiment was repeated 3 times. The percentage of cell survival was calculated according to the following equation:

(2) $$\begin{array}{c} \text{Survival}\;\text{fraction}\left(\%\right)=\left[\frac{\text{O}.\text{D}.\;\text{of}\;\text{treated}\;\text{cell}}{\text{O}.\text{D}.\;\text{of}\;\text{control}\;\text{cells}}\right]\times 100. \end{array}$$

### 2.5. Estimation of the Total Phenolic, Flavonoid, and Flavonol Contents

In this study, the total phenolic, flavonoid, and flavonol contents of crude methanol extracts, *n*-butanol, and aqueous fractions were measured according to the methods described by Abdel-Hameed, 2009 [14].

The total phenolic content of plant extracts was determined using Folin-Ciocalteu's reagent (FCR). 100 *μ*L of each sample solution (100 *μ*g/mL) and also 100 *μ*L of gallic acid (100 *μ*g/mL) were mixed with 500 *μ*L of the FCR and 1.5 mL of 20% sodium carbonate. The mixture was shaken thoroughly and brought up to 10 mL using distilled water. The mixture was allowed to stand for 2 h. Then the absorbance at 765 nm was determined against a blank that contained all reagents without the samples or the gallic acid at the same conditions. All determinations were carried out in triplicates. The total phenolic content was expressed as the number of equivalents of gallic acid (GAE).

The flavonoid content was determined by aluminium chloride method using rutin as a reference compound. 100 *μ*L of each sample solution (0.001 g/mL) was mixed with 100 *μ*L of 2% aluminum trichloride in ethanol and a drop of acetic acid and then diluted with ethanol to 5 mL. The absorption at 415 nm was read after 40 min. Blanks were prepared from all reagents without the samples. The absorption of the standard quercetin solution (0.1 mg/mL) in methanol was measured under the same conditions. All determinations were carried out in triplicates. The amount of flavonoids in plant extracts in quercetin equivalents (QE) was calculated by the following formula:

(3) $$\begin{array}{c} X=\frac{A-m_{o}}{A_{o}-m}, \end{array}$$

where *X* is the flavonoid content, mg/g plant extract in QE, *A* is the absorption of plant extract solution, *A*_*o*_ is the absorption of the standard quercetin solution, *m* is the weight of plant extract (g), and *m*_*o*_ is the weight of quercetin in the solution (mg).

The content of flavonols was determined by using quercetin as a reference compound. 1 mL of each sample solution (0.001 g/mL) was mixed with 1 mL aluminium trichloride (20 mg/mL) and 3 mL sodium acetate (50 mg/mL). The absorbance at 440 nm was read after 2.5 h. The absorption of the standard quercetin solution (0.5 mg/mL) in methanol was measured under the same conditions. All determinations were carried out in triplicates. The amount of flavonols in plant extracts in quercetin equivalents (QE) was calculated by the same formula used in flavonoids (3).

### 2.6. LC-ESI-MS Analysis

#### 2.6.1. Preparation of Standard and Sample Solutions

Ten standard stock solutions, catechin (500 *μ*g/mL), taxifolin (500 *μ*g/mL), quercetin-3-O-*β*-D-glucoside-6-gallic acid (500 *μ*g/mL), rutin (500 *μ*g/mL), quercetin 3-O-*β*-D-glucoside (500 *μ*g/mL), quercetin 3-O-*α*-L-rhamnoside (500 *μ*g/mL), kaempferol 3-O-*β*-D-glucoside (500 *μ*g/mL), kaempferol 3-O-*α*-L-rhamnoside (500 *μ*g/mL), quercetin (500 *μ*g/mL), and apigenin (500 *μ*g/mL), were prepared in HPLC grade solvent mixture of acetonitrile/methanol/water (1 : 1 : 2; v/v/v) and filtered using membrane disc filter (0.45 *μ*m). For example, solution (5 mg/mL) of all samples will prepared in HPLC grade solvent mixture of acetonitrile/methanol/water (1 : 1 : 2; v/v/v) and filtered using membrane disc filter (0.45 *μ*m).

#### 2.6.2. ESI-MS Direct Infusion

Direct infusion method using ESI-MS (Waters 3100) in negative ion mode was performed to get ESI(−ve)-MS fingerprinting for fresh and dry Taif roses. The analytical conditions for injection include the injection of all samples (5 mg/mL) directly to the ion source by means of a syringe pump at flow rate (0.02 mL/min) for ten minutes. The analytical conditions for mass spectrophotometer were capillary voltage (3 kV), cone voltage (30 and 70 eV), desolvation temperature (350°C), desolvation gas flow (700 L/h), cone gas flow (50 L/h), and source temperature (150°C). Mass spectra were scanned in ESI negative mode in the range between *m*/*z*  50 and 1000. Maslynx 4.1 software was used for data analysis. 

#### 2.6.3. LC-ESI-MS Conditions

LC-ESI-MS analysis system consists of HPLC (Waters Alliance 2695) and mass detector (Waters 3100). The mobile phases were prepared daily by filtering through 0.45 *μ*m membrane disc filter and degassed by sonication before use. The mobile phase for gradient elution consists of two solvents: solvent A (0.1% formic acid (FA) in water) and solvent B (0.1% FA in acetonitrile/methanol (1 : 1; v/v). The linear gradient profile was as follows: 95% A (5 min), 95–90% A (15 min), 90–50% A (50 min), 50–95% A (60 min), and 95% A (65 min). The injection volume was 20 *μ*L. The flow rate (0.6 mL/min) was splitted 1 : 1 before MS interface. The negative ion mode parameters were as follows: capillary voltage 3 kV, cone voltage 70 eV, source temperature 150°C, desolvation temperature 350°C, cone gas flow 50 L/h, and desolvation gas flow 700 L/h. Spectra was recorded in the ESI negative mode between *m*/*z* 50 and 1000. Peaks and spectra were processed using Maslynx 4.1 software. Known peak was identified by comparing its retention time (*t*_*R*_) and mass spectrum with a known standard. Unknown peak was tentatively identified by comparing its retention time and mass spectrum with literatures. 

#### 2.6.4. Calibration Curve and Sample Analysis

Each standard compound was chromatography using the previous analytical conditions. For each standard, the retention time and mass spectrum were determined. From each individual standard stock solution, a mixed stock solution containing ten analytes were prepared and diluted to appropriate different concentrations for establishing calibration curves. For quantitative analysis, six different concentrations of a mixed stock solution containing ten analytes were injected. A calibration curve was obtained by plotting the peak area versus the concentration of each standard. Chromatograms of samples obtained were analyzed using Maslynx 4.1 software based on the comparison of retention times of the samples with those of the standards for qualitative analysis and calibration curve for quantitative analysis.

### 2.7. Statistical Analysis

**Figure 1:**
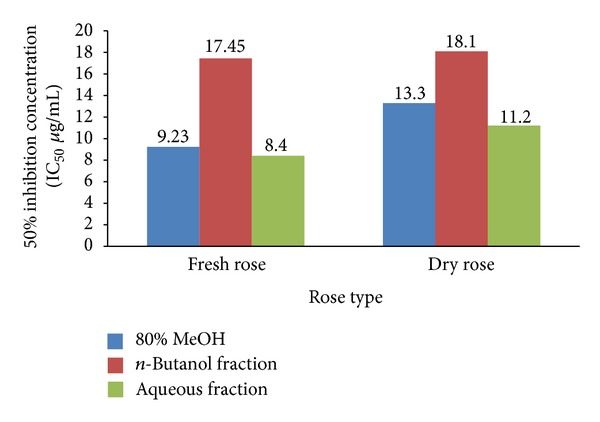
Cytotoxic activity expressed by IC_50_ (*μ*g/mL) of 80% methanol extracts, *n*-butanol, and aqueous fractions for both fresh and dry Taif rose toward human liver carcinoma cell line (HepG2). IC_50_ calculated from survival curve by plotting the percent of survival fraction against different concentrations of sample.

All determinations in Tables 1 and 2 were carried out in triplicate and presented as mean ± SD using SPSS 13.0 program. One-way ANOVA test followed by Duncan's test (*P* < 0.05) was used to analyze the differences among numbers per column. The SC_50_ values were calculated by probit-graphic interpolation for six concentration levels. The IC_50_ values in Figure 1 were calculated from survival curve of the tumor cell line by plotting the percent of survival fraction against different concentrations of sample. Correlation analyses were carried out using the correlation and regression by Microsoft Excel program.

## 3. Results and Discussion

The total phenolic contents and biological activity were reported in some *Rosa* species [12, 15–17]. Previous studies of *Rosa* species relate to the characterization of phenolic compounds by HPLC-UV(DAD) and LC-ESI-MS hyphenated techniques [6, 18–22]. According to our knowledge, until now there are no reports on the LC-MS study of Taif rose either fresh or dry. In this work, the fresh and dry Taif rose were biologically investigated for their antioxidant and anticancer activity. Phytochemical investigations included estimation of total phenolics, flavonoids, and flavonols with LC-ESI-MS analysis.

### 3.1. Antioxidant Activity

Free radicals and reactive oxygen species have been proposed to induce cellular damage and to be involved in several human diseases such as cancer, arteriosclerosis, and inflammatory disorders as well as in aging processes [23]. Recently, interest has increased considerably in finding naturally occurring antioxidants for use in foods, cosmetics, or medicinal materials to replace synthetic antioxidants, which are being restricted due to their carcinogenicity [24]. Dietary and herbal formulations which have free radical scavenging potential have gained important in treating such chronic diseases. Many reports attributed the antioxidant properties of vegetables, fruits, and medicinal plants to its contents of phenolic compounds [25–28].

The antioxidant activity of 80% methanol extracts, *n*-butanol, and aqueous fractions for both fresh and dry rose were chemically estimated by three methods as shown in Table 1. The results obtained from these methods can serve as a significant indicator for the antioxidant activity for samples. Both 80% methanol extracts for fresh and dry rose exhibited radical scavenging activity on DPPH^∙^ with SC_50_ values 12.18 and 12.24 *μ*g/mL, respectively. The SC_50_ values nearly equal the half value of standard ascorbic acid. On the other hand, the *n*-butanol fractions for both fresh and dry rose exhibited high radical scavenging activity nearly equal the standard ascorbic acid with SC_50_ values 5.86 and 7.19 *μ*g/mL, respectively, while the aqueous fractions showed very weak activity >100 *μ*g/mL. The determination of total antioxidant activity using phosphomolybdenum method showed that methanol and *n*-butanol fractions for both fresh and dry rose had high antioxidant capacity with values range 354.87–542.45 mg ascorbic acid equivalent/g extract, whereas the aqueous fractions showed weak activity. The methanol and *n*-butanol fractions for both fresh and dry rose exhibited high reducing power activity range 248.46–432.17 mg ascorbic acid equivalent/g extract, whereas the aqueous fractions showed weak activity. It is noticed that the partitioning of crude methanol extracts using organic and aqueous solvents plays important role in separation and concentration of the antioxidant compounds in organic fraction rather than the aqueous fractions. The result of this study was found in agreement with previous studies on the antioxidant activities of some *Rosa* species [6, 12, 17, 21].

### 3.2. Anticancer Activity

Cancer is a complicated group of diseases characterized by the uncontrolled growth and spread of abnormal cells, and the mortality that results from the common forms of cancer is still unacceptably high [29]. Hepatocellular carcinoma (HCC) is the third most common cause of cancer death in the world [30]. Cancer treatment through chemotherapy has serious limitation including resistance for cancer cells to these chemicals [31]. Thus, it is urgent to find more and more safe new compounds that kill cancer cells. Drug discovery from medicinal plants has played an important role in the treatment of cancer. Most new clinical applications of plant secondary metabolites and their derivatives over the last half century have been applied towards combating cancer [32]. Two plants derived natural products, Paclitaxel and Camptothecin, were estimated to account for nearly one-third of the global anticancer market in total annually in 2002 [33].

Figure 1 shows the cytotoxic effect of crude 80% methanol extracts, *n*-butanol, and aqueous fractions for both fresh and dry rose (IC_50_) against human hepatocellular carcinoma cell lines (HepG2) using the sulforhodamine B (SRB) method. The SRB method, which was developed by Skehan et al. (1990) [13] remains one of the most widely used methods for *in vitro* cytotoxicity screening. It has been widely used for drug-toxicity testing against different types of cancerous and noncancerous cell lines [34]. All samples showed high cytotoxic activity toward HepG2 with IC_50_ ranges 9.23–17.45 *μ*g/mL. According to the National Cancer Institute guideline, an extract and/or a compound with IC_50_ values <20 *μ*g/mL is considered active [35]. All Taif rose samples showed IC_50_ fall within this value; thus these samples are considered as promising anticancer agents. It is noticed that the crude 80% methanol extracts and aqueous fractions for both fresh and dry roses exhibited cytotoxic activity higher than the *n*-butanol fractions, therefore there are more than phytochemical classes responsible for the anticancer activity. In spite of the *n*-butanol fractions showing anticancer activity lower than the other fractions, they still fall within the range of NCI criteria. This result was in accordance with previous reports on the anticancer activity of some *Rosa* species [36, 37].

### 3.3. Total Phenolic, Flavonoid, and Flavonol Contents

Many reports attributed the biological properties of roses to its high contents of phenolic compounds [6, 12, 16, 36–38]. The total phenolic, flavonoid, and flavonol components of 80% methanol extracts, *n*-butanol, and aqueous fractions for both fresh and dry rose were estimated (Table 2). The *n*-butanol extracts for both fresh and dry rose has the highest contents (186.84 and 177.99 mg/g GAE, resp.) followed by methanol extracts (61.54 and 49.38 ± 1.27 mg/g GAE, resp.), while the aqueous fractions showed the least amount (7.73 and 7.74 mg/g GAE, resp.). Table 2 showed that the total flavonoids and flavonols of *n*-butanol fractions for both fresh and dry roses showed the highest content (63.18 and 65.59 mg/g QE for flavonoids and 34.46 and 40.51 mg/g QE for flavonols) followed by 80% methanol extracts (30.94 and 24.94 mg/g QE for flavonoids and 21.01 and 14.20 mg/g QE for flavonols), whereas aqueous fractions showed the least amount. It was noticed that there is little difference between the fresh and dried roses in the amounts of total phenolics, flavonoids, and flavonols which may be attributed to the presence of small amount of water in fresh roses that increase the polarity of extraction solvent (80% MeOH) during extraction process. Phenolic compounds especially tannins are highly polar compounds and more extracted with highly polar solvent mixtures. 

### 3.4. Correlation between the Total Phenolic, Flavonoid and Flavonol Contents with the Antioxidant and Anticancer Activity

The correlation coefficient between the total antioxidant capacity monitored by phosphomolybdenum method and the total phenolic, flavonoid, and flavonol contents of 80% methanol extracts, *n*-butanol, and aqueous fractions for both fresh and dry roses was determined. Linear correlation appeared between the total antioxidant capacity and the total phenolic, flavonoid, and flavonol contents with strong correlation coefficient (*R* ^2^ = 0.909 for total phenolics, *R* ^2^ = 0.960 for total flavonoids and *R*² = 0.928 for total flavonols). These results are in good accordance with previous studies that showed that high total phenolic content increases the antioxidant activity. The antioxidant activity of the phenolic compounds was attributed to its redox properties, which allow them to act as reducing agents, hydrogen donators, and singlet oxygen quenchers and have also metal chelating properties [14, 39]. The correlation coefficient between the anticancer activities (mortality percent at 25 *μ*g/mL extract) monitored by SRB method and the total phenolic, flavonoid, and flavonol contents 80% methanol extracts, *n*-butanol, and aqueous fractions for both fresh and dry roses was determined. No correlation appeared between the anticancer activity and the total phenolic, flavonoid, and flavonol contents. It could be concluded that the phenolic compounds which constitute the major phytochemical class of Taif rose play an important role and are responsible for antioxidant activity, while the cytotoxic activity was found to be associated with the occurrence of other nonphenolic compounds.

### 3.5. LC-ESI-MS Analysis

Hyphenated HPLC-MS technique is an important method used for identifying complex mixtures, especially the phenolics in the crude extract or its fraction found in the plant, either by using standard compounds (target identification) or by comparing mass spectrum obtained with literatures (tentative identification) [6, 21, 40–43]. This method is useful to avoid replication, safe time, and money used in isolation and identification of known compounds. In this part the different extracts of fresh and dry Taif roses were subjected to HPLC-MS analysis. 

**Figure 2:**
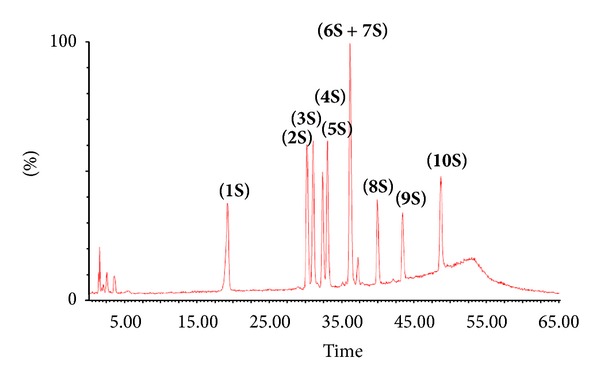
Total ion chromatogram of ten phenolic compound standards using LC-ESI negative mass spectrometry: Catechin **(S1)**; taxifolin **(S2)**; quercetin-3-glcucose-(1→6)-gallic acid **(S3)**; rutin **(S4)**; quercetin 3-O-*β*-D-glucoside **(S5)**; quercetin 3-O-*α*-rhamnoside **(S6)**; kaempferol 3-O-*β*-D-glucoside **(S7)**; kaempferol 3-O-*α*-rhamnoside **(S8)**; quercetin **(S9)**; and Apigenin **(S10)**.

After several trials to obtain good separation of the ten standard phenolic compounds mixture by LC-MS (Figure 2), nine sharp peaks were obtained. Good separated eight peaks which correspond to catechin **(S1)**, taxifolin **(S2)**, quercetin-3-O-*β*-D-glucoside-(1→6)-gallic acid **(S3)**, rutin **(S4)**, quercetin 3-O-*β*-D-glucoside **(S5)**, kaempferol 3-O-*α*-L-rhamnoside **(S8)**, quercetin **(S9)**, and apigenin **(S10)** were obtained. On the other hand, the remaining two standard compounds, quercetin 3-O-*α*-L-rhamnoside **(S6)** and kaempferol 3-O-*β*-D-glucoside **(S7)**, appeared at the same retention time. The MS spectrum of the first half of the peak is characteristic of **(S6)**, whereas the second half is characteristic of **(S7)**. A Calibration curve for each compound in the standard mixture was obtained, and the correlation coefficient revealed a good linearity response for method (Table 3).

The mass spectrum for both methanol and aqueous fractions obtained by direct infusion of samples into the negative ion mode ESI-MS showed the presence of a major peak at *m*/*z* 191 which was previously detected and identified in other *Rosa* species as quinic acid [21]. The ESI(−ve)-MS fingerprinting by direct infusion for both methanol and *n*-butanol fractions represents many peaks at *m*/*z*: 169, 183, 191, 301, 435, 447, 463, 483, 593, 599, 615, 635, 785, and 967. The previous peaks may be characterized as gallic acid and its methyl derivatives, quinic acid, flavonoid compounds, and hydrolysable tannins. The aqueous fractions showed peaks at *m*/*z*: 132; 179; 191; 293; 341; 391; 533. These peaks are characterized by their high polarity and may be attributed to organic acid (e.g., major peak at *m*/*z* 191; quinic acid), carbohydrate compounds, and unknown highly polar compounds.

**Figure 3:**
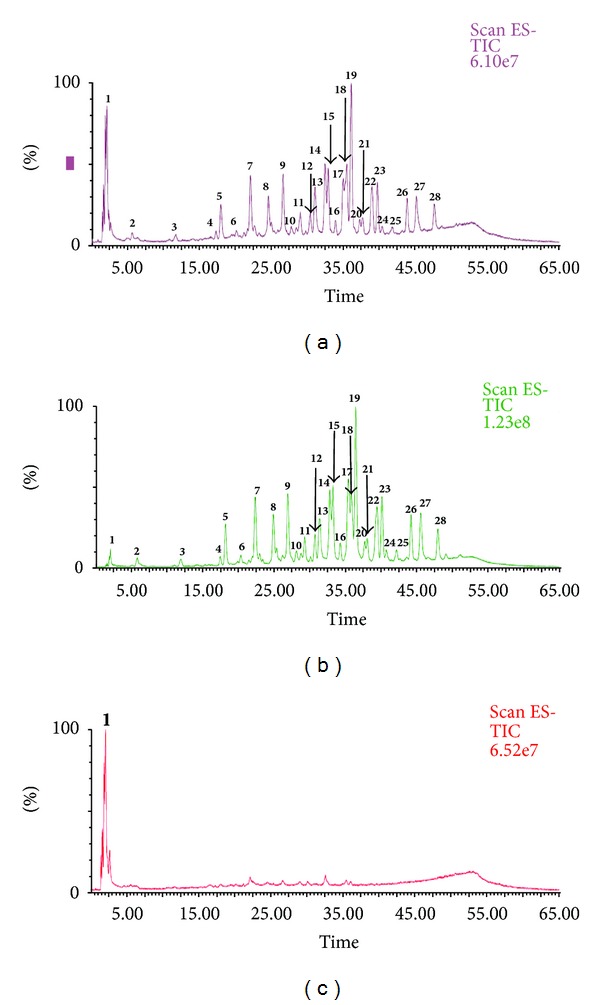
Total ion chromatograms (TIC) of 80% methanol extract (a), *n*-butanol (b), and aqueous (c) fractions of fresh Taif rose.

**Figure 4:**
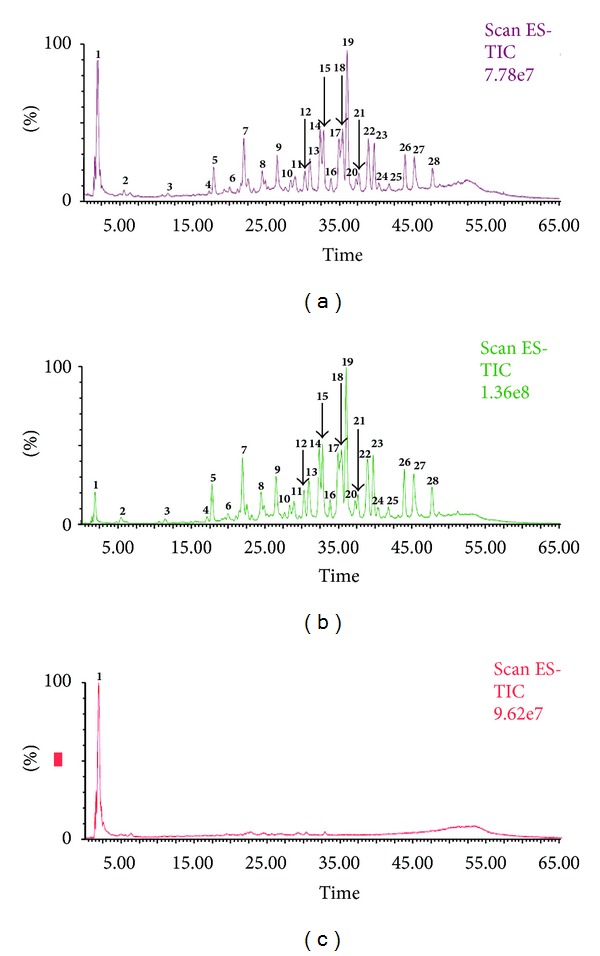
Total ion chromatograms (TIC) of 80% methanol extract (a), *n*-butanol (b), and aqueous (c) fractions of dry Taif rose.

**Figure 5:**
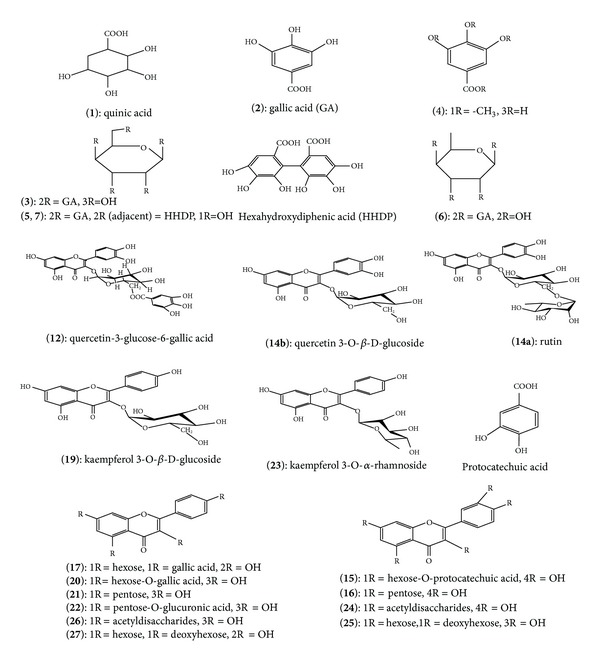
Chemical structures of compounds identified and tentatively identified from fresh and dry Taif rose.

After optimizing the LC separation method of standards, the samples were injected in LC-MS under the same conditions. Figures 3 and 4 represent the total ion chromatograms (TIC) of samples obtained from LC-MS. The spectra extracted from each chromatogram were investigated to identify or tentatively identify the components of each sample. The mass analysis of all chromatograms revealed the presence of one organic acid (quinic acid); 2 gallic acid and its mono-methyl derivative; 9 hydrolysable tannins which exhibited strong polarity and shorter retention time under reversed-phase chromatographic conditions; 16 flavonol glycosides of quercetin and kaempferol aglycones; and 1 unknown non phenolic compounds.  Figure 5 represents the chemical structures of compounds identified and tentatively identified from fresh and dry Taif rose. 

The molecular mass of sugar units in glycosides was calculated from the difference mass of molecular ion peaks, such that a difference of 132 indicates pentose (xylose/arabinose); a difference of 146 indicates deoxyhexose (rhamnose); a difference of 162 mass units indicates a hexose (glucose/galactose); a difference of 176 indicates glucuronic acid. Known peaks were identified by comparing their *t*_*R*_ and mass spectra with standards analyzed under the same analytical conditions, whereas unknown peaks were tentatively or postulated identified by comparing their *t*_*R*_ and mass spectra with literatures. By comparing, the TIC and mass spectrum of the standard compounds with the methanol, *n*-butanol, and aqueous fractions of fresh and dry Taif rose, five compounds were identified and quantified using calibration curve for each standard (Tables 4 and 5). The identified compounds which are quercetin-3-glucose-(1→6)-gallic acid **(12)**; rutin **(14a)**; quercetin 3-O-*β*-D-glucoside **(14b)**; kaempferol 3-O-*β*-D-glucoside **(19)**; and kaempferol 3-O-*α*-rhamnoside **(23)** were found. Traces of compound quercetin 3-O-*α*-rhamnoside are found within the peak **19**. The four remaining standard compounds, catechin **(S1)**; taxifolin **(S3)**; quercetin **(S9)**; and apigenin **(S10)**, were not detected. The six identified compounds were previously identified in some *Rosa* species [6, 21, 22]. Taxifolin was not detected in our work or in previous reports, while catechin, quercetin, and kampferol in addition to luteolin aglycons were detected in some *Rosa* species but in our work they were not detected [19, 21, 44]. The unknown peaks were tentatively identified based on their *t*_*R*_ and mass spectrum comparison with literatures and quantified from the calibration curve of similar standard compounds (Table 5). Compound **1** that showed *m*/*z* at 191 with fragments at *m*/*z* 172, 127, 93, and 85 was tentatively identified as quinic acid [41, 45]. This compound was also identified in other *Rosa* species [21]. Compound **2** showed molecular ion peak with *m*/*z*  169 which is characteristic of gallic acid by comparison of its mass spectra with literatures [42, 46]. This compound was previously identified in some *Rosa* species including Taif rose [6, 15, 21]. Compound **3** showed mass spectrum with *m*/*z* 483, 331, 313, 169, and 125 which is characteristic of digalloyl hexose isomer [21, 44, 47]. This compound was also identified in other *Rosa* species [21, 44]. Compound **4** has molecular ion peak at *m*/*z* 183 with fragments 169 and 124, which is characteristic to mono-methyl gallic acid derivative [48–50]. Peaks **5** and **7** showed the same molecular ion peak at *m*/*z* 785 and similar fragments. These two compounds were tentatively identified as HHDP-digalloyl hexose isomers [40, 42, 44, 51]. On the other hand, peak **6** showed a molecular ion peak at *m*/*z* 465 with fragments 313, 301, 169, and 125 which could be postulated to be digalloyl deoxyhexose isomer. The mass spectral data of peaks **8–11** and **13** were found in accordance with and associated to different isomers of unknown ellagitannins [40, 44, 47, 49, 51, 52]. Peak **15** showed molecular ion peak at *m*/*z*  599 with fragments 463, 300, 179, 169, and 151. The difference between ion at *m*/*z* 599 of 463, and 300 revealed the loss of two consecutive ions with 162 and 136 mass units which is characteristic to hexose sugar (galactose/glucose) and protocatechuic acid. Then this compound could be tentatively identified as quercetin-hexose-protocatechuic acid isomer, and it could be a good candidate for target separation and isolation. Peak **16** showed molecular ion peak at *m*/*z* 433 with fragments 301, 179 and 151 that is characteristic of quercetin-O-pentoside isomer [43, 44, 50]. Peaks **17** and **20** showed the same ion molecular peak at *m*/*z* 609 with fragments 447, 435, 284, 169, and 151 for peak **17** and fragments 447, 284, 197, 169, and 151 for peak **20**. The two compounds could be tentatively identified as kaempferol-O-hexose-O-gallic acid isomers in which compound **17** contains hexose and gallic acid in different situation. While compound **20** contains combined hexose-gallic acid [22], the mass spectrum of peak **18** showed that the presence of a mixture of two compounds is characteristic of kaempferol and quercetin disaccharides derivatives [22, 43, 50]. Peak **21** has a molecular ion peak at 417 with fragment at 284 in its mass spectrum. The difference between the molecular ion and its fragments (133 mass units) revealed the occurrence of pentose sugar (xylose/arabinose) attached to aglycon. Compound **21** could be tentatively identified as kaempferol-pentoside isomer [21, 22, 53]. Peak **22** with a molecular ion peak at *m*/*z* 593 with fragments 417 and 285 could give indication of the presence of pentose and glucuronic acid sugar moieties. This compound could be tentative identified as kaempferol-pentose-glucuronic acid isomer [47]. The two peaks **24** and **26** with molecular ion peak at 651 and 635 and its fragments were found in accordance with two compounds tentatively identified as quercetin-acetyl disaccharides and kaempferol-acetyl disaccharides [54, 55]. These two compounds were previously identified from the hydrodistilled petals from *Rosa damascena* [22]. The spectra of peaks **25** and **27** showed deprotonated molecular ion peaks [M−H]^−^at *m*/*z* 609 and 593. The spectrum of peak **25** showed fragments 463, 447, and 301, whereas spectrum of peak **27** has fragments 447, 430, and 284. The two compounds were found to be characteristic of quercetin disaccharides and kaempferol disaccharides isomers, respectively, in which the two sugar moieties attached with the aglycon in two different situations [22, 43, 46]. The last peak **28** with molecular ion peak at 604 showed different fragments of phenolic compound, and then this unknown compound could be tentatively identified as non phenolic compound.

## 4. Conclusions

The results of this study provide evidence that the 80% methanol extracts, *n*-butanol, and remaining aqueous fractions for both fresh and dry Taif roses have high antioxidant and anticancer activities. The antioxidant properties were found to be associated with percentage of total phenolics in each extract, whereas no association between anticancer activity and total phenolics appeared. The phytochemical analysis for fresh and dry Taif roses using modern hyphenated technique including high performance liquid chromatography coupled by electrospray ionization mass spectrometry (HPLC-ESI-MS) indicates the presence of phenolic compounds belonging to hydrolysable tannins and flavonol glycosides of quercetin and kaempferol aglycones. From these results, the methanol extracts and its *n*-butanol and aqueous fractions for both fresh and dry Taif rose could be used as preventive and therapeutic effective natural agents for diseases in which free radicals are involved after more *in vivo* and *in vitro* studies.

## Figures and Tables

**Table 1:** DPPH free radical scavenging activity, total antioxidant capacity, and reducing power activity of 80% methanol extracts, *n*-butanol, and aqueous fractions for both fresh and dry Taif rose.

Extract	DPPH free radical scavenging activity		Total antioxidant capacity (mg equivalent to ascorbic acid/g extract)^3^	Reducing power activity (mg equivalent to ascorbic acid/g extract)^4^
	SC_50_ (*μ*g/mL)^1^	(mg ascorbic acid equivalent/g extract)^2^		
Fresh roses				
80% MeOH	12.18 ± 0.07^c^	478.59 ± 2.79^a^	357.41 ± 6.58^b^	248.46 ± 2.70^b^
*n*-Butanol fraction	5.86 ± 0.07^a^	994.53 ± 11.90^c^	542.45 ± 9.56^d^	432.17 ± 2.13^e^
Aqueous fraction	>100^d^	—	166.03 ± 15.36^a^	100.54 ± 2.57^a^
Dry roses				
80% MeOH	12.24 ± 0.1^c^	476.21 ± 3.77^a^	354.87 ± 17.96^b^	278.80 ± 13.88^c^
*n*-Butanol fraction	7.19 ± 0.08^b^	810.96 ± 9.52^b^	504.43 ± 22.92^c^	396.72 ± 6.13^d^
Aqueous fraction	>100^d^	—	157.16 ± 5.8^a^	94.06 ± 1.77^a^
Ascorbic acid	5.83 ± 0.24^a^	—	—	—

Values of SC_50_, total antioxidant capacity reducing power activity, were expressed as mean of triplicate determinations ± standard deviation (*n* = 3). Values in the same column followed by a different letter (a–e) are significantly different (*P* < 0.05, ANOVA).

^1^SC_50_: concentration in *μ*g/mL required scavenging the DPPH radical (100 *μ*g/mL) by 50%. SC_50_ was calculated by probit-graphic interpolation for six concentration levels.

^2^Radical scavenging activity expressed by mg ascorbic acid equivalent/g extract.

^3^Antioxidant capacity monitored by the phosphomolybdenum method expressed by mg ascorbic acid equivalent/g extract.

^4^Reducing power activity expressed by mg ascorbic acid equivalent/g extract.

**Table 2:** Total amount of phenolic, flavonoid, and flavonol compounds of 80% methanol extracts, *n*-butanol, and aqueous fractions for both fresh and dry Taif rose.

Extract	Total phenolics (mg gallic acid equivalent/g extract)^1^	Total flavonoids (mg quercetin equivalent/g extract)^2^	Total flavonols (mg quercetin equivalent/g extract)^3^
Fresh roses			
80% MeOH	61.54 ± 3.88^c^	30.94 ± 0.39^c^	21.01 ± 0.55^c^
*n*-Butanol fraction	186.84 ± 6.94^e^	63.18 ± 0.76^d^	34.46 ± 0.58^d^
Aqueous fraction	7.73 ± 2.21^a^	3.61 ± 0.34^a^	3.08 ± 0.10^a^
Dry roses			
80% MeOH	49.38 ± 1.27^b^	24.94 ± 1.25^b^	14.20 ± 0.59^b^
*n*-Butanol fraction	177.99 ± 7.25^d^	65.59 ± 0.82^e^	40.51 ± 0.85^e^
Aqueous fraction	7.74 ± 1.91^a^	3.74 ± 0.17^a^	2.81 ± 0.13^a^

Values of SC_50_, total antioxidant capacity reducing power activity, were expressed as mean of triplicate determinations ± standard deviation (*n* = 3). Values in the same column followed by a different letter (a–e) are significantly different (*P* < 0.05, ANOVA).

^1^Total phenolics expressed by mg gallic acid equivalent/g extract.

^2^Total flavonoids expressed by mg quercetin equivalent/g extract.

^3^Total flavonols expressed by mg quercetin equivalent/g extract.

**Table 3:** Method validation data for ten phenolic compounds by RP-HPLC-ESI-MS.

Peak number	Compounds	Sample loading linearity range (*μ*g)	Regression equation	Correlation coefficient (*r*)
**S1**	Catechin	0.1–0.8	*y* = 9*E* + 06*x* + 2*E* + 06	0.878
**S2**	Taxifolin	0.1–0.8	*y* = 2*E* + 07*x* + 121352	0.998
**S3**	Quercetin-3-glucose-(1 → 6)-gallic acid	0.05–0.4	*y* = 4*E* + 07*x* + 181397	0.990
**S4**	Rutin	0.05–0.4	*y* = 3*E* + 07*x* − 37568	0.997
**S5**	Quercetin 3-O-*β*-D-glucoside	0.05–0.4	*y* = 4*E* + 07*x* + 209464	0.995
**S6 **&** S7**	Quercetin 3-O-*α*-rhamnoside + Kaempferol 3-O-*β*-D-glucoside	0.1–0.8	*y* = 3*E* + 07*x* + 1*E* + 06	0.998
**S8**	Kaempferol 3-O-*α*-rhamnoside	0.05–0.4	*y* = 2*E* + 07*x* + 123327	0.997
**S9**	Quercetin	0.025–0.2	*y* = 4*E* + 07*x* − 191266	0.998
**S10**	Apigenin	0.025–0.2	*y* = 5*E* + 07*x* + 573546	0.996

**Table 4:** Peak assignment, molecular weight (MW), molecular ion (M^−^), mass ion fragments, and tentative identification of compounds detected in 80% methanol extract, *n*-butanol, and aqueous fractions of fresh and dry Taif rose by LC-ESI(−ve)-MS.

Peak number	MW	*m*/*z*		Tentative identification
		M^−^	Fragments	
**1**	192	191	127, 93, 85	Quinic acid
**2**	170	169	125, 79	Gallic acid
**3**	484	483	331, 313, 169, 125	Digalloyl hexose
**4**	184	183	169, 147, 124, 78	Methyl gallic acid derivative
**5**	786	785	633, 615, 483, 301, 169, 125	Digalloyl DHHP hexose
**6**	466	465	313, 301, 169, 147, 125	Digalloyl deoxyhexose
**7**	786	785	633, 615, 483, 331, 313, 301, 169, 125	Digalloyl DHHP hexose
**8**	968	967	785, 765, 667, 505, 301, 183, 169	Unknown ellagitannin
**9**	968	967	785, 765, 633, 615, 483, 451, 301, 182, 169, 125	Unknown ellagitannin
**10**	938	937	783, 657, 465, 301, 169, 125	Unknown ellagitannin
**11**	968	967	785, 765, 639, 450, 314, 301, 169, 147, 124	Unknown ellagitannin
**12**	616	615	463, 313, 301, 169	Quercetin-3-glucose-(1 → 6)-gallic acid^a^
**13**	938	937	785, 766, 615, 313, 301, 183, 169, 125	Unknown ellagitannin
**14a**	610	609	463, 301	Rutin^a^
**14b**	464	463	301, 229, 179, 150	Quercetin 3-O-*β*-D-glucoside^a^
**15**	600	599	463, 300, 179, 169, 151	Quercetin-hexose-protocatechuic acid
**16**	434	433	301, 151, 179	Quercetin-O-pentose
**17**	600	609	447, 435, 284, 169, 151	Kaempferol-hexose-gallic acid
**18**	610/594	609/593	435, 433, 301, 285, 169, 151	Quercetin/Kaempferol derivatives
**19**	448	447	284, 179, 151	Kaempferol 3-O-*β*-D-glucoside^a^
**20**	600	599	447, 285, 197, 169, 151	Kaempferol-O-hexose-O-gallic acid
**21**	418	417	284, 197, 227	Kaempferol-O-pentose
**22**	594	593	417, 285, 197, 151, 147	Kaempferol-O-pentose-O-glucuronic acid
**23**	432	431	284, 255, 227	Kaempferol 3-O-*α*-rhamnoside^a^
**24**	652	651	609, 447, 301, 147	Quercetin acetyldisaccharides
**25**	610	609	463, 447, 301, 147	Quercetin-O-hexose-O-deoxyhexose
**26**	636	635	487, 285	Kaempferol acetyldisaccharides
**27**	594	593	447, 430, 285, 151	Kaempferol-O-hexose-O-deoxyhexose
**28**	605	604	582, 462, 342	Unknown non-phenolic compound

Standard compounds				
**S1**	290	289	244, 221, 150, 136, 123	Catechin
**S2**	304	303	284, 274, 217, 179, 151	Taxifolin
**S3**	616	615	463, 313, 301, 271, 169, 151, 147	Quercetin-3-glucose-6-gallic acid
**S4**	610	609	463, 301, 179, 151, 147	Rutin
**S5**	464	463	300, 271, 254, 179, 151	Quercetin 3-O-*β*-D-glucoside
**S6 **	448	447	300, 270, 179, 151	Quercetin 3-O-*α*-rhamnoside
**S7**	448	447	284, 179, 151	Kaempferol 3-O-*β*-D-glucoside
**S8**	432	431	248, 254, 227, 198, 147	Kaempferol 3-O-*α*-rhamnoside
**S9**	302	301	179, 151	Quercetin
**S10**	270	269	225, 199, 159, 151, 117	Apigenin

^a^Compounds identified by comparison with standards.

**Table 5:** Quantity of compounds detected in 80% methanol extract, *n*-butanol, and aqueous fractions of fresh and dry Taif rose.

Peak number	Compound	Fresh rose (mg/g extract)			Dry rose (mg/g extract)		
		80% MeOH	*n*-BuOH	Aqueous	80% MeOH	*n*-BuOH	Aqueous
**1**	Quinic acid^a^	1546329	351441	1749393	2076361	731132	26452471
**2**	Gallic acid	3.22	6.31	—	2.84	5.39	—
**3**	Digalloyl hexose^1^	0.09	0.31	—	0.04	0.16	—
**4**	Methyl gallic acid derivative^2^	3.57	7.6	—	2.1	5.3	—
**5**	Digalloyl HHDP hexose^1^	1.03	2.84	—	1.17	2.80	—
**6**	Digalloyl deoxyhexose^1^	0.09	0.22	—	0.11	0.19	—
**7**	Digalloyl HHDP hexose^1^	2.18	5.35	—	2.60	5.32	—
**8**	Unknown ellagitannin^1^	1.57	4.06	—	1.12	2.66	—
**9**	Unknown ellagitannin^1^	2.52	6.30	—	1.72	3.85	—
**10**	Unknown ellagitannin^1^	0.22	0.80	—	0.49	0.40	—
**11**	Unknown ellagitannin^1^	0.75	1.56	—	0.87	1.44	—
**12**	Quercetin-3-glucose-6-gallic acid	1.33	1.38	—	0.64	1.43	—
**13**	Unknown ellagitannin^1^	2.06	4.07	—	1.61	3.50	—
**14a**	Rutin	0.10	0.17	—	0.10	0.24	—
**14b**	Quercetin 3-O-*β*-D-glucoside	1.90	3.93	—	2.28	4.72	—
**15**	Quercetin-glucose-protocatechuic acid^1^	1.46	3.62	—	1.93	3.64	—
**16**	Quercetin-pentoside^3^	0.21	0.65	—	0.29	0.78	—
**17**	Kaempferol-hexose-gallic acid^1^	1.27	4.77	—	1.70	3.33	—
**18**	Quercetin/Kaempferol derivatives^4^	2.81	5.88	—	3.43	6.66	—
**19**	Kaempferol 3-O-*β*-D-glucoside	7.50	16.21	—	9.15	17.60	—
**20**	Kaempferol-hexose-gallic acid^1^	0.14	0.31	—	0.22	0.43	—
**21**	Kaempferol-pentoside^5^	0.53	1.30	—	0.87	1.50	—
**22**	Kaempferol-pentoside glucuronic acid^4^	1.95	5.21	—	2.65	5.71	—
**23**	Kaempferol 3-O-*α*-rhamnoside	0.54	1.31	—	0.88	1.50	—
**24**	Quercetin acetyl disaccharides^4^	0.23	0.50	—	0.21	0.58	—
**25**	Quercetin disaccharides^4^	0.26	0.65	—	0.19	0.66	—
**26**	Kaempferol acetyl disaccharides^4^	1.27	3.28	—	1.50	3.44	—
**27**	Kaempferol hexoside-deoxyhexose^4^	1.46	3.94	—	1.59	3.74	—
**28**	Unknown non-phenolic compound^a^	2776578	6225284	—	541747	7069559	

Quantified as ^1^quercetin-3-glucose-6-gallic acid; ^2^gallic acid; ^3^quercetin 3-O-*β*-D-glucoside; ^4^rutin, and ^5^kaempferol 3-O-*α*-rhamnoside (including molecular weight correction factor).

^a^Due to lake of similar or related standard compounds, the quantity is represented by area under curve.
